# Strategy to enhance lung cancer treatment by five essential elements: inhalation delivery, nanotechnology, tumor-receptor targeting, chemo- and gene therapy

**DOI:** 10.7150/thno.39816

**Published:** 2019-10-22

**Authors:** Olga B. Garbuzenko, Andriy Kuzmov, Oleh Taratula, Sharon R. Pine, Tamara Minko

**Affiliations:** 1 Department of Pharmaceutics, Ernest Mario School of Pharmacy, Rutgers, the State University of New Jersey, Piscataway, NJ 08854, USA; 2 Department of Pharmaceutical Sciences, College of Pharmacy, Oregon State University, Portland, OR 97201, USA; 3 Rutgers Cancer Institute of New Jersey, New Brunswick, NJ 08903, USA; 4 Departments of Pharmacology and Medicine, Rutgers, the State University of New Jersey, Piscataway, NJ 08854, USA; 5 Environmental and Occupational Health Science Institute, Piscataway, NJ 08854, USA

**Keywords:** Imaging, LHRH peptide, suppression of EGFR-TK signaling pathways, pool of siRNAs, lung cancer cells with different resistance to gefitinib

## Abstract

Non-Small Cell Lung Carcinoma (NSCLC), is the most common type of lung cancer (more than 80% of all cases). Small molecule Tyrosine Kinase (TK) Inhibitors acting on the Epidermal Growth Factor Receptors (EGFRs) are standard therapies for patients with NSCLC harboring EGFR-TK inhibitor-sensitizing mutations. However, fewer than 10 % of patients with NSCLC benefit from this therapy. Moreover, even the latest generation of EGFR inhibitors can cause severe systemic toxicities and are ineffective in preventing non-canonical EGFR signaling. In order to minimize and even overcome these limitations, we are proposing a novel multi-tier biotechnology treatment approach that includes: (1) suppression of all four types of EGFR-TKs by a pool of small interfering RNAs (siRNAs); (2) induction of cell death by an anticancer drug, (3) enhancing the efficiency of the treatment by the local inhalation delivery of therapeutic agents directly to the lungs (passive targeting), (4) active receptor-mediated targeting of the therapy specifically to cancer cells that in turn should minimize adverse side effects of treatment and (5) increasing the stability, solubility, and cellular penetration of siRNA and drug by using tumor targeted Nanostructured Lipid Carriers (NLC).

**Methods**: NLCs targeted to NSCLC cells by a synthetic Luteinizing Hormone-Releasing Hormone (LHRH) decapeptide was used for the simultaneous delivery of paclitaxel (TAX) and a pool of siRNAs targeted to the four major forms of EGFR-TKs. LHRH-NLC-siRNAs-TAX nanoparticles were synthesized, characterized and tested *in vitro* using human lung cancer cells with different sensitivities to gefitinib (inhibitor of EGFR) and *in vivo* on an orthotopic NSCLC mouse model.

**Results**: Proposed nanoparticle-based complex containing an anticancer drug, inhibitors of different types of EGFR-TKs and peptide targeted to the tumor-specific receptors (LHRH-NLC-siRNAs-TAX) demonstrated a favorable organ distribution and superior anticancer effect when compared with treatment by a single drug, inhibitor of one EGFR-TK and non-targeted therapy.

**Conclusion**s: The use of a multifunctional NLC-based delivery system substantially enhanced the efficiency of therapy for NSCLC and possibly will limit adverse side effects of the treatments. The results obtained have the potential to significantly impact the field of drug delivery and to improve the efficiency of therapy of lung and other types of cancer.

## Introduction

About 228,150 new lung cancer cases are expected to be diagnosed in 2019 in USA, accounting for more than 30% of all cancer diagnoses 1. In 2019, about 142,670 Americans are expected to die of lung and bronchus cancer, or more than 390 people per day. Non-Small Cell Lung Carcinoma (NSCLC) is the most common type of lung cancer, accounting for more than 84% of cases 1. The majority of NSCLC patients are diagnosed in the advanced or metastatic stage of the disease, when treatment options are limited to surgery, chemotherapy, few targeted- and immunotherapy. Consequently, the development of additional effective and safe approaches to treat this disease is vitally important. However, limited clinical efficiency, toxicity and development of resistance represent three critical barriers diminishing progress in the therapy of NSCLC.

Oncogenic receptor Tyrosine Kinase (TK) pathways, specifically Epidermal Growth Factor (EGF) pathways have been explored as targets for therapy of NSCLC, and EGF receptor (EGFR) inhibitors are currently used as first-line therapy options for a subset of patients with advanced stages of the disease 2. Monoclonal antibodies targeted to EGFR and small-molecule TK inhibitors (erlotinib, gefitinib, afatinib, dacomitinib, and osimertinib) responseshave extended the lives of lung cancer patients harboring EGFR-TKI-sensitizing mutations. However, most of these agents target only one type of EGFR, are effective only in a small fraction of patients (about 10%) with specific EGFR mutations (mostly deletions in exon 19 and nucleotide substitutions in exon 21) 3, and elicit numerous intrinsic and acquired resistance mechanisms (*e.g.*, T790M mutation) 4-7. As a result of intrinsic resistance, 20-30% of NSCLC patients with activating EGFR mutations do not respond to the treatment with EGFR-TK inhibitors and almost all patients with an initial positive response eventually develop acquired resistance within a relatively short time of treatment 6. Moreover, some EGFR-TK inhibitors demonstrate substantial toxicity in patients with different types of mutations 5.

**Figure 1:**
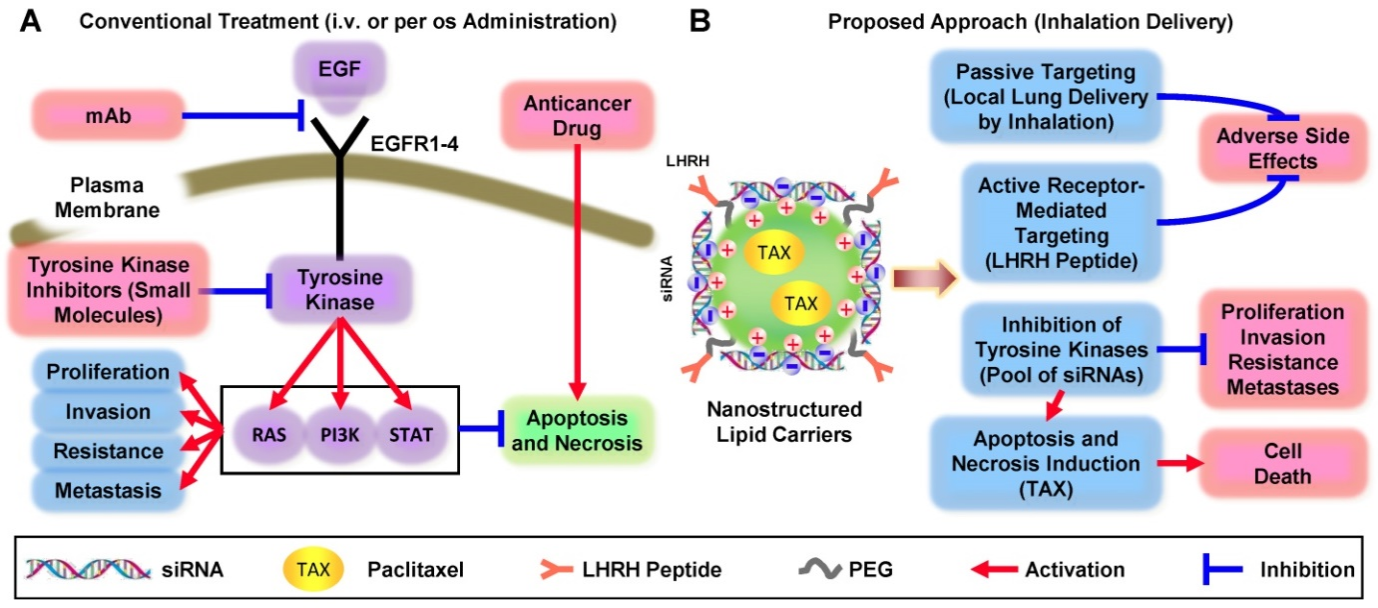
Comparison of conventional and non-traditional proposed approaches for treatment of non-small cell lung carcinoma.

Activation of EGF receptors (EGFR1, EGFR2, EGFR3, EGFR4) in response to ligands (*e.g.*, EGF, TGFα, and others) triggers several downstream signaling pathways via three main signaling branches: Rat Sarcoma (RAS), Phosphatidylinositol 3′-kinase (PI3K) and Signal Transducers and Activators of Transcription (STAT) (Figure 1A). Each EGFR family member has been implicated in driving NSCLC initiation and progression 8. The activation of these pathways ultimately inhibits apoptosis and necrosis and activates uncontrolled cellular proliferation, invasion, metastasis, and resistance.

Three major nonsurgical approaches are currently being used to treat NSCLC: (1) chemotherapy plus immunotherap; (2) monoclonal antibodies (mAb) specific to EGFR blocking EGF signaling pathway and (3) inhibition of EGFR-TK by small-molecule(s). The major disadvantages of chemotherapy by cytotoxic drugs include severe adverse side effects on healthy tissues and development of drug resistance. The disadvantages of EGFR-targeted therapy both by mAb and small-molecule TK inhibitors include: (1) specificity to only a single type of EGFR-TK (and therefore effectiveness only in small fraction of patients) and/or (2) side effects of treatment. To overcome these limitations, enhance the efficiency of NSCLC treatment, and prevent systemic toxicity, we are proposing an innovative multi-tier treatment approach (Figure 1B). This approach includes inhibition of an entire pool of TKs in NSCLC cells by genotherapy with multiple siRNAs combined with chemotherapy. To limit adverse side effects of such combinatorial geno- and chemotherapy, we will combine siRNAs and an anticancer drug in one multifunctional system and explore its delivery directly to the lung by inhalation. To further protect non-cancerous lung cells and target not only the primary tumor but also possible metastases, an entire system will contain a ligand (LHRH peptide) specific to extracellular receptors overexpressed in lung cancer cells 9. We hypothesized that the application of a such tumor-targeted, multifunctional approach will substantially enhance the efficiency of the treatment and limit adverse side effects of chemotherapy by simultaneous local delivery to the lungs (by inhalation) and specific to lung cancer cells targeted therapy. Such an approach should lead to the suppression of cancer cell proliferation, limitation of drug resistance, invasion, and metastasis by inhibition of EGFR-TK signaling pathways in combination with chemotherapy. The main objectives of the current research are to test the stated hypothesis, introduce an innovative approach to overcome existing critical barriers in the chemotherapy of NSCLC, and to develop a novel nanoscale-based technology to carry out a proof-of-concept of the proposed strategy. In the present study, we focused our efforts on lung adenocarcinoma (LUAD), a histological subtype accounting for 50% of NSCLC cases, because EGFR-targeted therapies have traditionally been most effective against ADC.

## Methods

### Materials

Precirol ATO 5 was generously provided by Gattefossé USA (Paramus, NJ). Soybean phosphatidylcholine (SPC), Paclitaxel (TAX), Squalene, Tween-80, Mannitol were purchased from Sigma Aldrich (St. Louis, MO). DSPE-PEG (1,2-distearoyl-sn-glycero-3-phosphoethanolamine-N-[amino(polyethylene glycol)-2000] were obtained from Avanti Polar Lipids (Alabaster, AL). XenoLight DiR obtained from Perkin Elmer (Akron, OH). A modified synthetic analog of Luteinizing Hormone-Releasing Hormone (LHRH) decapeptide (Gln-His-Trp-Ser-Tyr-DLys(D-Cys)-Leu-Arg-Pro) was synthesized according to our design by American Peptide Company, Inc. (Sunnyvale, CA). The sequence of native LHRH peptide, which is similar in human, mouse, and rat, was modified to provide a reactive amino group only on the side chain of a lysine residue, which replaced Gly at the position 6 to yield the superactive, degradation-resistant-Lys-6-des-Gly-10-Pro-9-ethylamide LHRH analog 10-13. The synthesized sequence of LHRH peptide is highly efficient for targeting of drug delivery systems specifically to the cancer tumors 9, 10, 14-17 and Cys residue do not influence the recognition process. The remmining materials were obtained from Sigma Aldrich (St. Louis, MO).

### Synthesis of Targeted Drug and siRNA Delivery System

Nanostructured Lipid Carriers (NLC) were developed by nanostructuring the lipid matrix in order to give more flexibility for modulation of drug release, increasing the drug loading and preventing its leakage 18-20. This was accomplished by mixing solid lipids with liquid lipids, which resulted in a less ordered lipid matrix with many imperfections that help to accommodate a higher amount of payload. The synthetic procedures were developed and tested in our laboratory in order to construct cationic NLC, incorporate TAX, and form complexes with anionic siRNAs.

**Drug Containing NLC.** NLC were prepared by a modified melted ultrasonic method 20. The lipid and aqueous phases were prepared separately in glass vials. Typically, the lipid phase consisted of 100 mg Precirol ATO 5 (solid lipid), 100 mg Squalene (liquid lipid) and 5 mg soybean phosphatidylcholine (lipophilic emulsifier). In the preliminary studies to test our design strategy, Paclitaxel (TAX) was used as a model drug to be encapsulated into NLC. To prepare drug-loaded NLC, 5mg of TAX dissolved in 500 µL of DMSO were added to the hot lipid phase under stirring. Aqueous phase was prepared by dissolving 250 mg Tween-80 (surfactant) and 25 mg DOTAP (cationic lipid) in 10 mL of DI water. In order to prepare PEG coated NLC, 10 mg DSPE-PEG2000 with or without the LHRH peptide were additionally added to the aqueous phase. Previously we studied the influence of targeted and non-targeted PEG polymers with different molecular weight, also, nanocarriers with different composition, size and shape on the efficiency of cellular penetration and overall therapeutic effect. Our data showed that PEG2000 is the most suitable for the linkage of LHRH peptide to the lipid nanoparticles 9, 14, 21, 22. XenoLight DIR-labeled NLC was prepared with the addition of XenoLight DIR dye (Perkin Elmer, Akron, OH) into the mixture of lipids as previously described 20. Both phases were maintained for 15 min at a temperature above the melting point of the lipid (80 °C) in an oil bath under magnetic stirring (1,000 rpm). The hot lipid phase then was added slowly to the aqueous solution under same temperature and magnetic stirring conditions (1,000 rpm, 80 °C). The melted lipid mass was dispersed in the aqueous solution using a high-speed homogenizer (PRO200, PRO Scientific, Inc.) at 18,000 rpm for 10 min to form a hot O/W emulsion. The crude emulsion was then treated by ultrasonication (TELSONIC, 60 W output) for 5 min at 80 °C. The hot emulsion was cooled at 4 °C, maintaining the mechanical stirring at 1,000 rpm for 1 h. Finally, the NLC were purified by extensive dialysis against water (10,000 MWCO). Dialyzed nanoparticles (100-150 nm) were collected and subjected to lyophilization using mannitol as lyoprotectant.

**Preparation of siRNA-NLC Complexes**. The pool of siRNAs, siGENOME Human EGFR (1956) siRNA - SMARTpool (Cat.No M-003114-03-0050, Dharmacon, Lafayette, CO) containing four or more EGFR-specific siRNA duplexes 23 was used to suppress all four EGFRs and limit uncontrolled proliferation of cancer cells, vascular invasion, resistance, and metastases. In some experiments, DY-547 (siGLO Red) labeled siRNA (Ambion, Inc, Austin, TX) replaced pool of siRNAs to visualize siRNA delivered into the cells by a fluorescence or confocal microscope as well as *in vivo* imaging. According to the validated protocol, siRNA solution was added to the purified nanoparticles dissolved in water to obtain final nucleic acid concentration of 1 µM. The mixture was gently vortexed and incubated at 25 °C for 30 min to ensure complete siRNA binding to NLS. In order to study a siRNA complexation, 1 µM siRNA was added to 10 µg, 20 µg, 30 µg, 40 µg, 50 µg, and 80 µg of cationic NLC. The mixture of NLC and siRNA was vortexed and incubated at room temperature for 30-60 min to allow siRNA to form complexes with the NLC. Next, the amount of free siRNA was visualized by a submarine gel electrophoresis with one well representing 1 µM free siRNA and the rest of the wells representing the above mentioned complexes. The gel was imaged using a Gel Logic 440 Imaging System (Kodak, Rochester, NY).

### Characterization of Drug and siRNA Delivery System

Nanoparticle size, shape, charge and loading efficiency were analyzed using Atomic Force Microscopy (AFM, Nanoscope IIIA, Veeco Digital Instruments, Ford, PA), Malvern ZetaSizer Nanoseries (Malvern Instruments, UK), and HPLC (Waters Corporation, Milford, MA). Methods of such analyses were previously developed and validated in our laboratory 15, 20, 22, 24-29.

### siRNA Serum Stability

Serum stability of both free siRNA and modified siRNA complexes before and after nebulization was investigated by incubating each formulation at 37 ºC with equal volume of human serum to give 50% serum concentration. At each predetermined time interval, (0, 5, 15, 30min, 1, 2, 3, 4, 5, 6, 7, 24 and 48 h) 50 μL of the mixture were removed and stored at -20ºC until gel electrophoresis was performed. In order to release siRNA from the complexes for gel electrophoresis, each sample was treated with 25 mM of reduced glutathione and 100 μM of PMAA. The aliquots from different incubation time periods were loaded onto 4% NuSieve 3:1 Reliant agarose gels in 1×TBE buffer (0.089 M Tris/Borate, 0.002 M EDTA, pH 8.3; Research Organic Inc., Cleveland, OH) and subjected to submarine electrophoresis. The gels were stained with EtBr, digitally photographed, and scanned using Gel Documentation System 920 (NucleoTech, San Mateo, CA).

### Cellular Internalization

In order to visualize a drug, TAX, Oregon Green™ 488 Conjugate (Catalog number: P22310, Grand Island, NY) was used to prepare an aliquot of drug-loaded NLC. Different components of delivery system were labeled with different fluorescent dyes: NLC - near-infrared fluorescence, TAX - green fluorescence and siRNA - red fluorescence, were prepared as described above. A549 adenocarcinomic human basal epithelial (alveolar type II pneumocytes) non-small cell lung cancer (NSCLC) cells were plated in 6 well plates and treated with the fluorescently labelled NLC-TAX-siRNA formulations for three hours. The cells were then visualized using a confocal microscope Leica G-STED SP8 (Olympus America Inc., Melville, NY).

### Cytotoxicity, Apoptosis Induction and Immune Response

The toxicities of the developed formulations were compared with a commercially used EGFR inhibitor gefitinib in three types of human lung cancer cells with different resistance to the drug. The following types of NSCLC cell lines were used: (1) NCI-H1781 gefitinib-insensitive (EGFR2-mutant); (2) A549 (no EGFR-TK mutations) with moderate sensitivity to gefitinib; and (3) NCI-H3255 gefitinib sensitive (EGFR1-L858R mutant). Cytotoxicity and apoptosis induction were analyzed by a modified 3-(4,5-Dimethylthiazol-2-yl)-2,5-diphenyltetrazolium bromide (MTT) assay and cell death detection ELISA Plus kit (Roche, Nutley, NJ), respectively. The cells were seeded on a 96 well plate at 10,000 cells per well with 100 µl of growth media in each well and placed in the incubator for 24 hours. After 24 hours, the media was removed and the cells were treated with varying concentrations of above indicated substances. The concentrations of tested substances were prepared using a serial dilution (1:2) to make 12 different working solutions for the cytotoxicity measurement.

For apoptosis induction study, cells were incubated for 72 h with 1 µM of gefitinib (the most effective concentration 30) or 0.3 ng/ml of NLC-siRNAs-TAX (IC_50_ dose for A549 cells determined by cellular cytotoxicity experiments). The control cells were incubated with fresh media. Previously validated in our laboratory MTT and apoptosis induction measurement protocols were used 10, 16, 28, 31, 32. In order to test a toxicity of NLC, apoptosis induction was measured in different organs (liver, kidney, spleen, heart, lung, brain) of mice after injection of saline (control) and NLC intravenously or by inhalation.

In order to examine an immune response for the developed complex system, human peripheral blood lymphocytes (PBL) were incubated with non-targeted and targeted NLC containing siRNA within 8 days and concentration of immunoglobulin G (IgG) was measured using immunoperoxidase assay (Immunology Consultants Laboratory, Inc., Newberg, OR).

### Expression of EGFR mRNA

Mouse lungs were extracted, trachea and mainstream bronchi were separated, lungs were frozen and homogenized as described 33. RNA was isolated using RNeasy kit (Qiagen, Valencia, CA) according to manufacturer's protocol. First-strand cDNA was synthesized with Ready-To-Go You- Prime First-Strand Beads (Amersham Biosciences, Piscataway, NJ) with 1 μg of total cellular RNA (from 107 cells) and 100 ng of random hexadeoxynucleotide primer (Amersham Biosciences, Piscataway, NJ). Then, the reaction mixture was immediately subjected to Quantitative Reverse Transcription Polymerase Chain Reaction - QRT-PCR (Applied Biosystems, Inc., Foster City, CA) using previously developed and validated sets of EGFR-TK primers 34, 35.

### *In Vivo* Lung Cancer Model, Imaging and Treatment

All animal experiments were carried out according to the protocol approved by Institutional Animal Care and Use Committee (IACUC). Nude mice were purchased from the approved commercial vendor (Taconic, Germantown, NY). All aspects of the program for procurement, conditioning/quarantine, housing management, veterinary care and disposal of carcasses followed the guidelines set forth in the NIH Guide for the Care and Use of Laboratory Animals. Animals were housed in the AALAC (American Association for Laboratory Animal Science) - accredited animal facility with free access to food and water. Veterinary care was supplied by Rutgers University Lab Animal Services. Orthotopic lung cancer mouse model was created by intratracheal instillation of A549 lung cancer cells as previously described 20, 21, 25, 36-39. The progression of tumor growth was monitored using bioluminescent and magnetic resonance (MRI) imaging. Luciferase transfected cancer cells were visualized in live anesthetized animals using in-vivo bioluminescence IVIS system (Xenogen, Alameda, CA). Luciferin (150 mg/kg) was intraperitoneally administered 10-15 minutes before imaging. All imaging procedures were performed at special imaging facilities of the Rutgers University Molecular Imaging Center as previously described 16, 20, 25, 40, 41. Mice were anesthetized with isoflurane (4 % for induction of anesthesia and 1-2 % for maintenance) using XGI-8 Gas Anesthesia System (Xenogen, Alameda, CA) for all imaging procedures. MRI was performed using a 1 T M2™ whole body scanner (Aspect Imaging, Shoham, Israel). Images (repetition time 2607 ms, echo time 44 ms) were recorded in Fast Spin Echo sequence. At a spatial resolution of 312 μm, the tumors were coronal imaged in a single section through the mouse body using an image matrix of 256 × 256, a field of view of 80 mm^2^, and 4 excitation. Magnetic resonance signal and tumor volume were calculated using VivoQuant 1.21 software (Invicro, Boston, MA).

**Figure 2:**
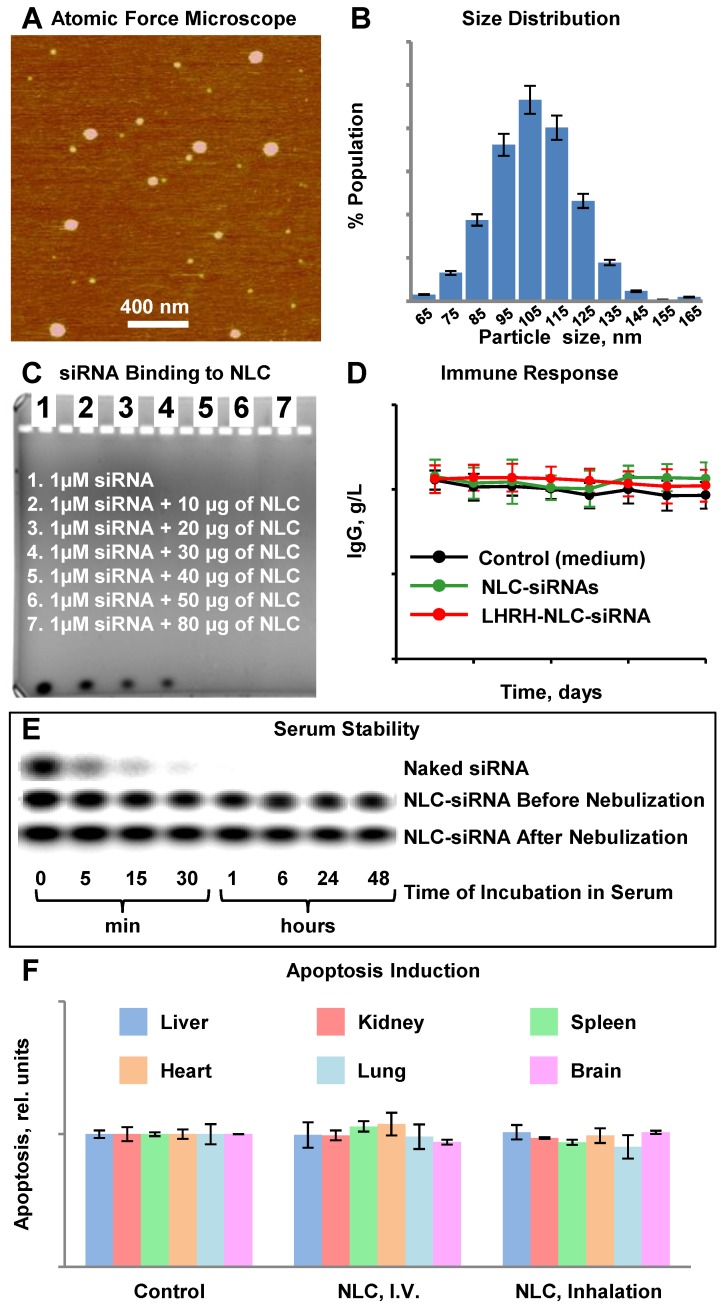
Characterization of Nanostructured Lipid Carriers (NLCs). A - Representative atomic force microscope images of NLC-siRNA complexes. B - Histogram of NLC-siRNA size distribution. C - Typical image of siRNA in agarose gel stained with ethidium bromide incubated with different amount of NLC. D - IgG secretion *in vitro* in supernatants of peripheral blood lymphocytes cultured with medium (control) and pool of siRNAs delivered by non-targeted and LHRH-targeted NLC. E - The human serum stability of non-condensed naked siRNA (upper panel), and the engineered siRNA nanoparticles before (middle panel) and after (lower panel) nebulization. F - Apoptosis induction in different organs in animals treated with saline (control), NLC by intravenous injection (I.V.) and by inhalation. The enrichment of histone-associated DNA fragments (mono- and oligonucleosomes) per gram tissue in different organs were measured. Values in control animals were set to unit 1, and the degree of apoptosis was expressed in relative units. F - Apoptosis induction Means ± SD are shown.

Organ distribution of NLC was studied as previously described 20, 25, 42. Briefly, aliquots of NLC were labeled with near-infrared fluorescent XenoLight DiR dye (Perkin Elmer, Akron, OH). A fluorescent dye was dissolved together with lipids in chloroform. Fluorescence of labeled with DiR NLCs were visualized 24 h after the intravenous administration or by inhalation using IVIS imaging system. Visible light and fluorescence images were taken and overlaid using the manufacturer's software to obtain composite images. The distribution of fluorescence in different organs (liver, kidney spleen, heart, brain and lungs) was analyzed using original software developed in our laboratory. Four-six weeks after the instillation of tumor cells, when the tumor in lungs reached a volume of about 40 mm^3^, mice were treated on days 0, 3, 7, 11, 14, 17, 21, and 24 with free non-bound TAX (intravenous injections), non-targeted NLC-siRNAs (inhalation), non-targeted NLC-TAX (inhalation), non-targeted NLC-siRNAs-TAX (inhalation), tumor targeted LHRH-NLC-siRNAs-TAX (inhalation). In order to deliver NLC-based system by inhalation, a Collison nebulizer (BGI, Inc., Waltham, MA) was connected to four-port nose-only exposure chambers (the chamber was made by CH Technologies, Westwood, NJ according to our design) (Figure 2). The uniformity of the nanoparticle size in the inhalation delivery was previously studied in details 43. We designed the installation and selected regime of inhalation to keep the inhalation dosage controllable and uniform from animal to animal with a minimal damage to the nanoparticles. The solution of NLC was aerosolized at a flow rate of 2 L/min and then diluted by an additional 2 L/min of air. It was found that aerosolization did not significantly change the shape and size of the nanoparticles 20. It was also shown that the aerosol distribution between the four ports of the chamber was homogenous 43. The dose of TAX in all drug-containing formulations was 2.5 mg/kg for the single administration. This dose corresponds to the maximum tolerated dose (MTD) of LHRH-NLC-TAX delivered by inhalation. The MTD was estimated in separate experiments based on animal weight change after the instillation of increasing doses of drug formulation as previously described 10, 20, 25, 44. The dose of siRNA was 170 μg/kg for the single administration. The dose of free non-bound TAX used for intravenous injection was also 2.5 mg/kg. Animal weight was monitored every day during the treatment period. After the treatment, all mice were anesthetized with isoflurane and euthanized. Changes in tumor volume were used as an overall marker for antitumor activity. After sacrificing the animals, lungs and other organs were excised, washed in ice-cold saline and kept frozen.

### Statistical Analysis

Data were analyzed using descriptive statistics, single-factor analysis of variance (ANOVA), and presented as mean values ± standard deviation (SD) from four to ten independent measurements. Ten animals were used in each experimental group. The comparison among groups was performed by the independent sample student's *t*-test. The difference between variants was considered significant if *P* < 0.05.

### Biological Variables

Age, weight, and underlying health condition for all experimental animals were similar in all experimental groups. To increase scientific rigor, we used animals of both sexes in the experiments since lung cancer affects both genders. No statistically significant difference was found between genders and, therefore, the data for both sexes were combined in one data set.

## Results

### Preparation and Characterization of Nanoparticle-Based Delivery System

The synthesized NLC had a distinct spherical shape and average diameter of 111.3 ± 20 nm (Figure 2A, B). Figure 2B demonstrates a size distribution of a large number of single nanoparticles and their aggregates in the solution measured by dynamic light scattering (DLS) technique. In contrast, the provided AFM image (Figure 2A) reveals only a tiny portion of nanoparticles from the sample in their dry state deposited on mica surface, and therefore, it may not represent the actual ratio of small to large nanoparticles in the sample. Consequently, the AFM image confirms a spherical shape of the prepared nanoparticles, and the DLS profile demonstrates the average hydrodynamic size and size distribution of nanoparticles in the sample. The measured average zeta-potential of the particles was about +45 mV. TAX loading efficiency of NLC was estimated to be about 98 %. According to the gel retardation assay (Figure 2C), disappearance of the siRNA band in the agarose gel after its incubation with 40 µg NLC indicating that all siRNA molecules were complexed by NLC, and therefore the loading efficiency of siRNA into NLC-based delivery system was 100% at this siRNA:NLC w/w ratio. The nanoparticles formed complexes with tested siRNA resulting in practically neutral composition of NLC-based delivery system. Binding of the anionic siRNA to the cationic NLC was studied using an agarose gel retardation assay (Figure 2C). Disappearance of the siRNA band in the agarose gel after its incubation with various amounts of NLC (10 µg, 20 µg, 30 µg, 40 µg, 50 µg, and 80 µg) confirms that 40 µg NLC is required to complex 1 µM (~0.33 µg) siRNA, which corresponds to weight NLC/weight siRNA ratio = 121. The resulting complexes were stable at 4 °C for at least two months and were effectively internalized by cancer cells. The average siRNA loading was about 2.5 nmole/mg of NLC. In order to estimate an immune response to siRNA incorporated in the NLC-based delivery system, human peripheral blood lymphocytes (PBL) were incubated with non-targeted and targeted NLC containing siRNA within 8 days and concentration of immunoglobulin G (IgG) was measured using immunoperoxidase assay. It was found, that both non-targeted and LHRH-targeted NLC loaded with siRNA did not induce an immune response in human PBL (Figure 2D). In addition, measurement of apoptosis induction *in vivo* in different organs (liver, kidney, spleen, heart, lung, brain) of mice treated with NLC administered by intravenous injection and inhalation did not show any signs of toxicity (Figure 2F).

### siRNA Serum Stability

The stabilization of siRNA in human serum against the nuclease degradation is essential for *in vivo* siRNA delivery. Therefore, to examine siRNA protection in the engineered siRNA nanoparticles from nuclease degradation, the complexes were incubated in the presence of 50% human serum at 37ºC. Gel electrophoresis experiment demonstrated that the degradation of free non-bound naked siRNA was observed 5 min after the incubation, while 1 h after the beginning of the experiment, siRNA was fully digested (Fig. 2E, upper panel). On the other hand, the complexation of siRNA with NLC effectively stabilized siRNA in human serum for the entire duration of the experiment (48 h, Fig. 2E, middle panel). Moreover, nebulization of siRNA complexed with NLC did not decrease siRNA stability and prevented its degradation by nucleases.

### Cellular Internalization of NLC-siRNA-TAX

**Figure 3:**
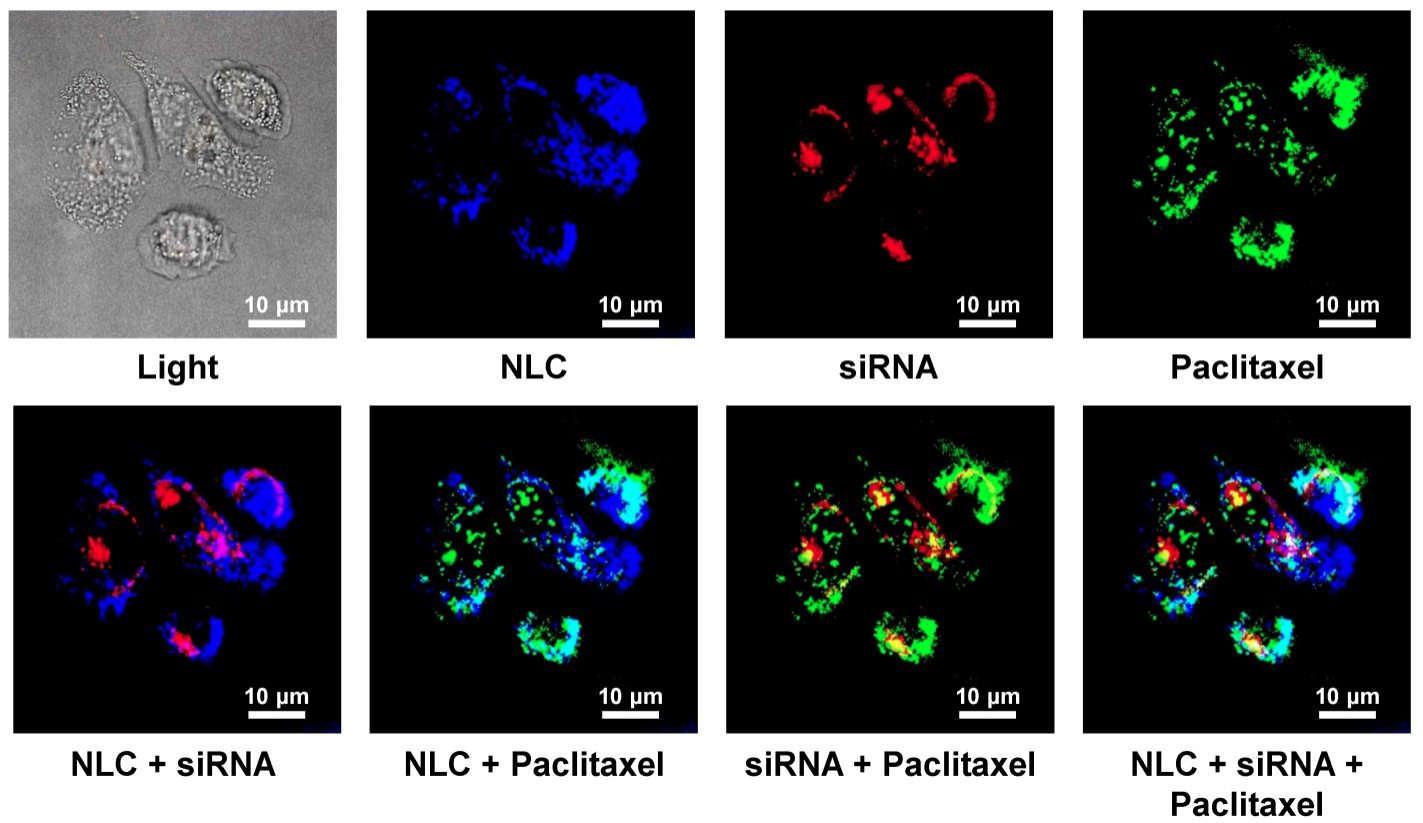
Cellular internalization of Nanostructured Lipid Carriers (NLC), siRNA and paclitaxel delivered by NLC (confocal microscope Leica G-STED SP8). A549 adenocarcinomic human basal epithelial (alveolar type II pneumocytes) non-small cell lung cancer (NSCLC) cells were incubated for 18 h with NLC (blue color represents near-infrared fluorescence) containing siRNA (red fluorescence) and paclitaxel (green fluorescence). Superimposition of red and blue colors gives pink color; superimposition of blue and green gives cyan color; superimposition of red and green colors gives yellow color; superimposition of red, green and blue colors gives white color.

Cellular Internalization of NLC, incorporated drug and siRNA was studied in human A549 NSCLC cells using a confocal microscope. Figure 3 shows representative fluorescence images of the cells incubated with NLC-siRNA-TAX complexes. Labeled NLC emitted near-infrared (visualized by a confocal microscopy as blue color), siRNA - red and paclitaxel green fluorescence. Superimposition of red and blue colors gives pink color; superimposition of blue and green gives cyan color; superimposition of red and green colors gives yellow color; superimposition of red, green and blue colors gives white color. We found that NLCs as well as their cargo, TAX and siRNA, successfully internalized into the lung cancer cells. Since fluorescence of siRNA and TAX was quenched in complexes, registered red and green fluorescence inside the cells reflected the release and localization of free non-bound siRNA and TAX inside cancer cells, respectively. It was found, that developed NLC-based delivery system effectively transferred siRNA and TAX and released them in cancer cells.

### Cytotoxicity and Competitive Inhibition by LHRH peptide

**Figure 4:**
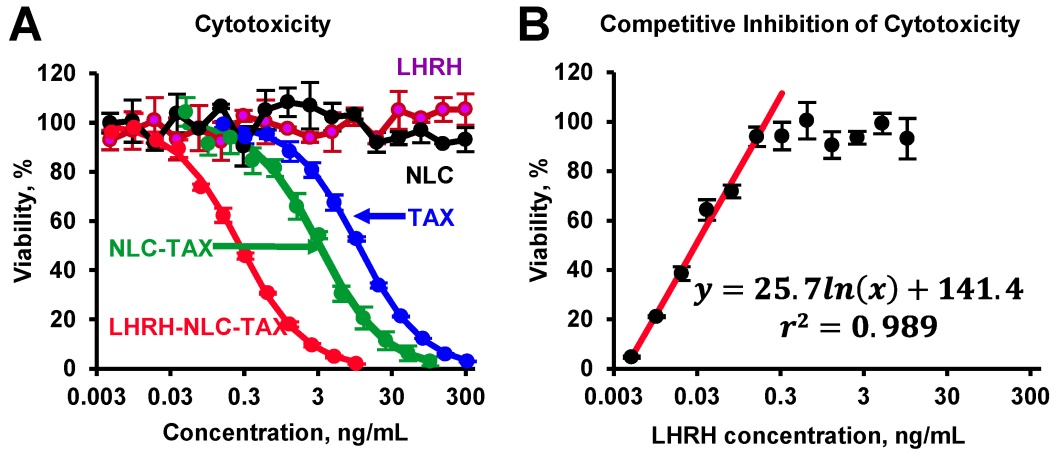
Cytotoxicity of different formulations in human A549 NSCLC cells (A) and competitive inhibition of cellular cytotoxicity of LHRH-NLC-TAX complex by free LHRH peptide (B). Means ± SD are shown.

The toxicity studies showed that the nanoparticles without siRNA and drug as well as LHRH peptide alone did not demonstrate any toxicity (Figure 4A). The delivery of TAX by non-targeted NLC substantially enhanced cytotoxicity of TAX against cancer cells. Furthermore, it was found that targeting of NLC-TAX using LHRH significantly increased the cytotoxicity of complexes in LHRH-positive lung cancer cells. To confirm that such an enhancement in cytotoxicity of LHRH-NLC-TAX system is associated with its binding to LHRH receptor, we incubated NSCLC cells with different concentrations of LHRH peptide (ligand for LHRH receptor) and then measured cytotoxicity of LHRH-NLC-TAX complex. Data obtained showed that LHRH receptor ligand limited cytotoxicity of LHRH-NLC-TAX in a concentration-dependent manner (Figure 4B).

**Figure 5:**
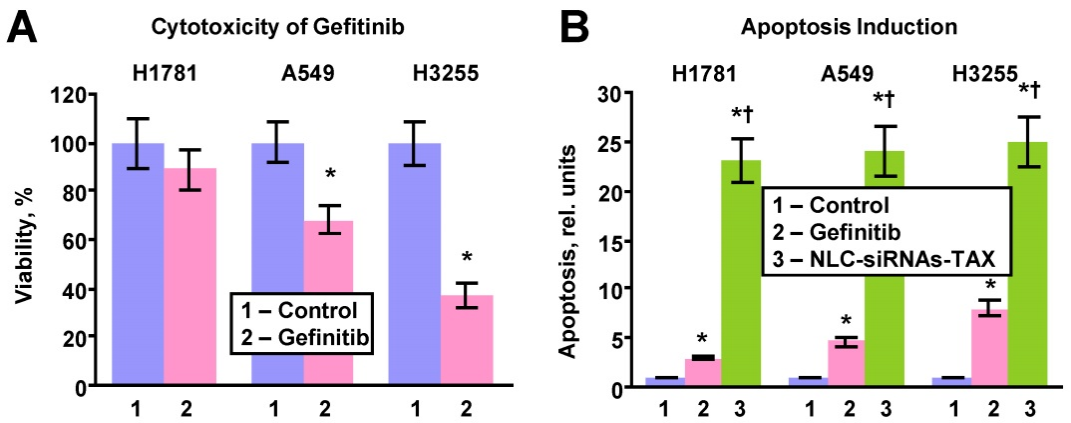
Cytotoxicity and apoptosis induction in human lung cancer cells with different sensitivity to gefitinib. H1781 gefitinib-insensitive (EGFR2-mutant), A549 (no EGFR-TK mutations) with moderate sensitivity to gefitinib and H3255 gefitinib sensitive (EGFR1-L858R mutant) cells were incubated within 24h with fresh media (control), gefitinib (1 μM) and NLC-siRNAs-TAX (0.3 ng/mL). A - Cytotoxicity. B - Apoptosis induction. Means ± SD are shown. *P < 0.05 when compared with control. ^†^*P* < 0.05 when compared with gefitinib.

The efficacy of killing lung cancer cells and apoptosis induction by the LHRH-NLC-TAX multifunctional complex system was compared with a traditional clinically used EGFR-TK inhibitor - gefitinib (Figure 5). In these experiments, three types of human lung cancer cells with different sensitivity to gefitinib were incubated for 72 h with the most effective concentration (1 µM) of gefitinib or IC_50_ dose of LHRH-NLC-TAX for A549 cells (0.3 ng/mL). It was found that H1781 cells were most resistant to gefitinib (Fig. 5A). In contrast, H3255 cells with mutated EGFR1-TK were the most sensitive to gefitinib. A549 NSCLC cells had a moderate sensitivity to gefitinib. The efficiency of apoptosis induction by the conventional drug (gefitinib) and NLC-siRNAs-TAX were compared (Fig. 5B). It was found that apoptosis induction by gefitinib correlated with its cytotoxicity, while NCL-siRNAs-TAX induced apoptosis with almost the same efficiency (*P* > 0.05) in all tested cell lines with differing sensitivity to gefitinib. Overall, NLC-siRNAs-TAX was 3-7 times more efficient when compared with gefitinib. Our data show that proposed approach utilizing complex gene- and chemotherapy (simultaneous delivery of TAX and siRNAs targeted to the pool of EGFR-TKs) effectively killed all types of human NSCLC cells (with and without mutations of EGFR-TKs).

### Evaluation of Orthotopic Lung Cancer Model

**Figure 6:**
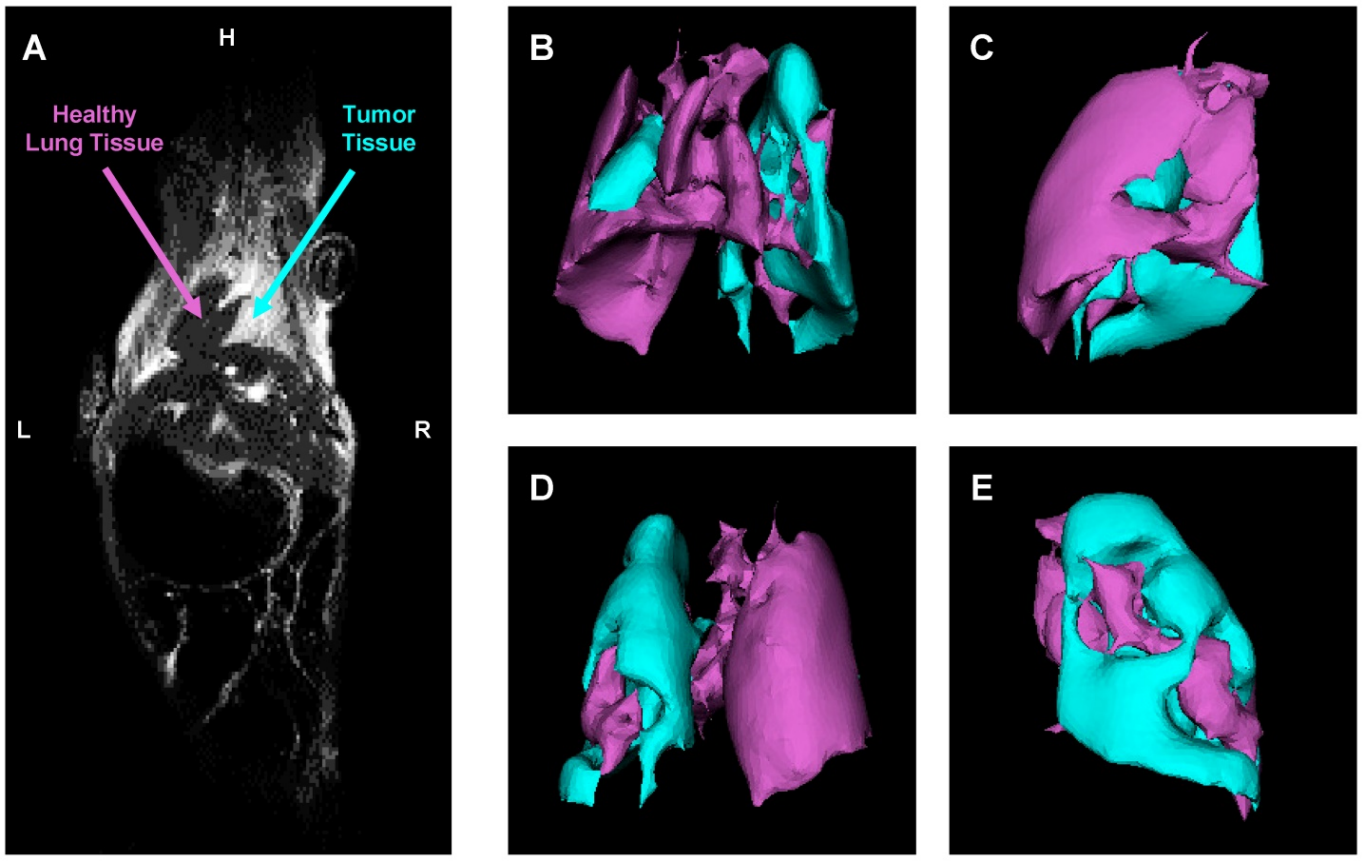
Evaluation of orthotopic model of human NSCLC in nude mice by a magnetic resonance imaging (MRI). A - Representative MRI image of a mouse with lung tumor. B-E - Computer analysis of representative MRI image of mouse lungs. Bright rich purple (orchid) color represents a healthy lung tissue, while cyan - tumor tissue (B - top; C - right; D - bottom; E - left).

**Figure 7:**
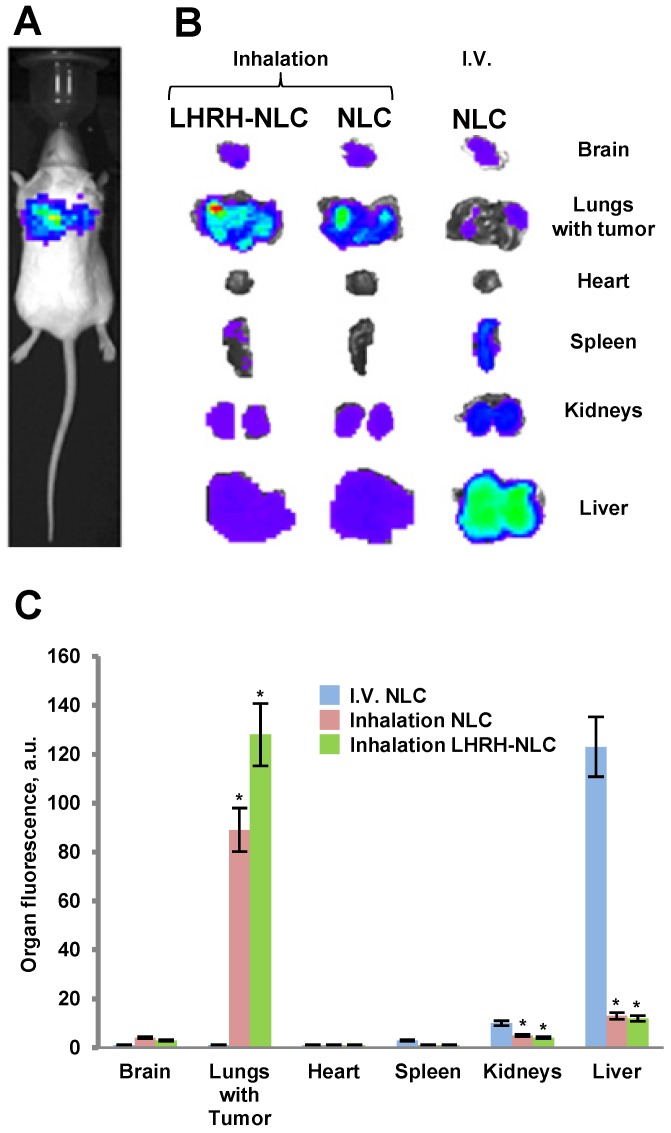
Distribution (optical imaging, IVIS) of non-targeted NLC and tumor-targeted LHRH-NLC in mice with orthotopic model of human lung cancer. A - A representative image of mouse with orthotopic model of lung cancer (human A549 NSCLC cells transfected with luciferase were inoculated intratracheally into the lungs of nude mice); B, C - Organ distribution of fluorescently labeled by DiR NLC (I.V. and inhalation administration) and LHRH-NLC (inhalation administration) in mice with lung tumor. Ten animals were used in each experimental group. Means ± SD are shown. *P < 0.05 when compared with I.V. injection.

An orthotopic mouse model of human lung cancer was developed in our lab according to a protocol approved by the Institutional IACUC. According to the protocol, NSCLC cancer cells (human A549) transfected with luciferase in a medium containing 5 μmole of EDTA were administered intratracheally to the mouse lung through a catheter. It was shown that slight disruption of the pulmonary epithelium or the surfactant layer by co-administration of either pancreatic elastase or EDTA allowed significantly better tumor engraftment 45. Suspension of cancer cells and this concentration of EDTA displayed no adverse side effects in test animals. Rapid growth of lung tumor occurred in 80% of animals. The progression of tumor growth was monitored using bioluminescent (IVIS) and magnetic resonance imaging systems. As an example of MRI image processing, a representative images of an entire mouse and mouse lungs with tumor are shown on Figure 6A-E. MRI was performed using a 1 T M2™ whole body scanner and images were recorded in Fast Spin Echo sequence. Magnetic resonance signal and tumor volume were calculated using VivoQuant 1.21 software. The analysis of lung images by VivoQuant software is shown on the Fig 6B-E images which show rotated representative image of lungs with tumor where a where a bright rich purple (orchid) color represents a healthy lung tissue, while cyan - tumorous tissue. Image analysis clearly shows the development of lung tumor and validates the orthotopic lung tumor model. Bioluminescence images of transfected NSCLC cells also support the development of lung cancer (Figure 7A).

### Biodistribution of Tumor Receptor-Targeted and Non-Targeted Delivery Systems

**Figure 8:**
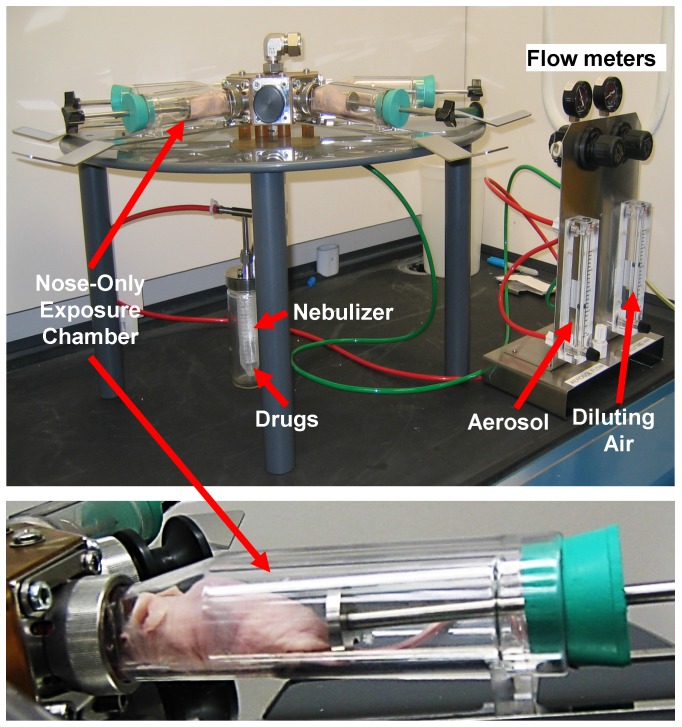
Instillation for inhalation delivery of drug and siRNA NLC-formulations.

Body distribution of the proposed LHRH-NLC system was studied and compared with those for non-targeted NLC delivered either intravenously or by inhalation. A previously constructed and tested four channel instillation for inhalation delivery of nanoparticle drugs 25, 43 was used in these experiments (Figure 8). The instillation allowed for a uniform distribution of inhaled substances among the four inhalation chambers contained anesthetized animals with a minimal influence on the shape and efficacy of inhaled substances.

The accumulation of fluorescently labelled targeted and non-targeted NLCs within different organs (lungs, liver, kidney, spleen, brain, heart) of mouse containing orthotopic lung tumor was studied using IVIS system in live anesthetized animals and their excised organs. A typical image of an entire animal and its organs treated with targeted and non-targeted NLCs is presented on Figures 7B, C along with calculated average distribution of tested nanocarriers. It was found that non-targeted NLC after intravenous injection accumulated predominately in the mouse liver and partially in kidney and spleen. Inhalation of such nanoparticles substantially changed their body distribution with a significant decrease in accumulation in the liver and efficient retaining in the lungs. Targeting of the NLCs specifically for tumor cells by the LHRH peptide significantly enhanced accumulation of LHRH-NLC nanoparticles in the lungs with tumor after inhalation delivery. It should be stressed that accumulation of tumor-targeted nanoparticles (LHRH-NLC) after inhalation is greater when compared not only with I.V. administration of these particles but also with non-targeted NLCs also delivered by inhalation. This phenomenon creates prerequisites for enhancing the anticancer activity of delivered biologically active anticancer agents and limit adverse side effects of the treatment.

### Efficacy of Inhalation Treatment of Lung Cancer using Nanoparticle-Based Tumor-Targeted Complex Delivery System

**Figure 9:**
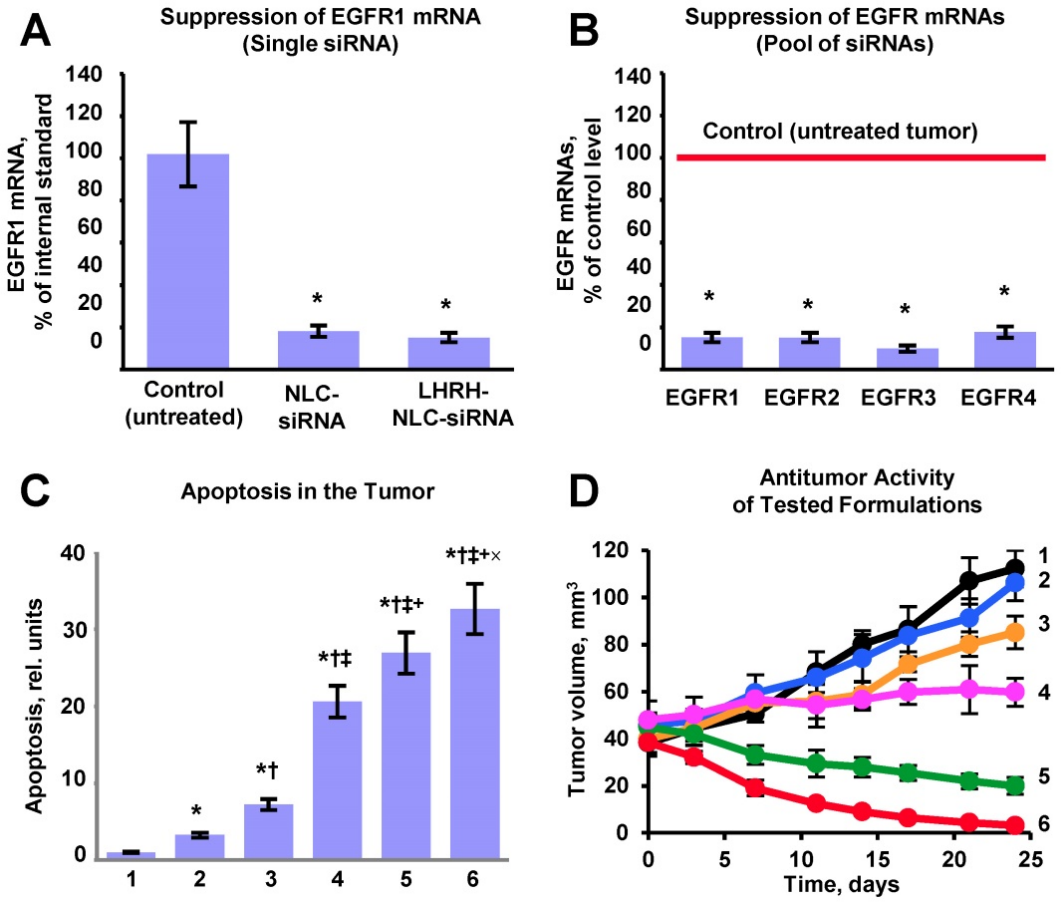
Evaluation of the proposed complex approach for treatment of mice with human non-small cell lung carcinoma. A - Suppression of targeted EGFR1-TK mRNA by siRNA delivered by non-targeted and targeted NLC. B - Suppression of four EGFR-TKs by the selected pool of siRNAs delivered by targeted LHRH-NLC-siRNAs-TAX system. C - Apoptosis induction in the lung tumor. D - Changes in lung tumor volume after beginning of treatment (mice were treated on days 0, 3, 7, 11, 14, 17, 21 and 24). The progression of tumor growth was monitored using bioluminescent and magnetic resonance imaging and tumor volume was calculated using software supplied with IVIS and magnetic resonance imaging systems. 1 - Control (untreated tumor); 2 - Free non-bound TAX (I.V. administration); 3 - Non-targeted NLC-siRNAs (inhalation); 4 - Non-targeted NLC-TAX (inhalation); 5 - Non-targeted NLC-siRNAs-TAX (inhalation); 6 - tumor targeted LHRH-NLC-siRNAs-TAX (inhalation). Means ± SD are shown. *P < 0.05 when compared with control;*^†^P* < 0.05 when compared with free TAX;*^‡^P* < 0.05 when compare with NLC-siRNAs;*^+^P* < 0.05 when compared with NLC-TAX*; ^×^P* < 0.05 when compared with NLC-siRNA-TAX.

Efficient accumulation of siRNAs and a chemotherapeutic drug in the lungs as well as their uptake by cancer cells does not automatically guarantee suppression of targeted mRNAs and corresponding proteins accompanied by effective killing of cancer cells. Consequently, we evaluated the efficiency of the suppression of all four variants of EGFR-TK receptors (Figures 9A, B), cell death induction in tumor cells (Figure 9C) and inhibition of tumor growth by the proposed NLC-based delivery system (Figure 9D). To this end, an expression of the most common variants of EGFR-TKs mRNAs was analyzed. Induction of apoptosis and/or necrosis was also studied in the same experimental setting. The correlation between the expression of studied proteins, degree of apoptosis and/or necrosis, and tumor suppression by the LHRH-NLC-siRNA-TAX with appropriate controls were analyzed. It was shown that targeted delivery of siRNA pool specifically to lung tumor cells effectively suppressed all four major forms of EGFR-TK receptors. Measurement of tumor volume at the end of the experiment showed that treatment of mice with TAX delivered intravenously decreased the lung tumor just in 1.1 times, treatment of mice by inhalation with NLC-siRNA shrank the tumor in 1.3 times, NLC-TAX - in 1.9 times, while the combination of NLC with siRNA and TAX decreased the tumor size in 5.6 times. It should be stressed that the combination of siRNA and TAX delivered together by inhalation using NLC shrank the volume of lung tumor significantly better when compared with either the drug or siRNA alone. It was found that lung tumor was almost completely eliminated after the treatment of mice with tumor targeted LHRH-NLC-siRNAs-TAX delivered by inhalation (Fig. 9D). Finally, we found that simultaneous targeted inhalation delivery of siRNAs with TAX (a combination of gene- and chemotherapy) resulted in killing of NSCLC cells, limiting and suppression of tumor growth with the efficiency that cannot be achieved when either therapy is applied alone/separately.

## Discussion

NLC incorporated positive characteristics of several different drug carriers used for siRNA and drug delivery including liposomes, solid lipid nanoparticles, polymeric nanoparticles and emulsions 46-50. NLC as carriers provide the following advantages when compared with other lipid carriers: (1) improved stability of encapsulated drugs; (2) controlled and/or targeted drug release; (3) high and enhanced content of payload(s); (4) ability to carry both drugs and nucleic acids; (5) ability to incorporate tumor-specific targeting ligand; (6) excellent biocompatibility (composed of biodegradable lipids); (7) easy sterilization and scale-up production for animal experiments and clinical trials; (8) water-based technology (avoiding potentially toxic organic solvents); and (9) less expensive than polymeric/surfactant based carriers. Previously, we performed studies to select the most appropriate carrier for inhalatory delivery of a drug and siRNA. Different nanocarriers (liposomes, micelles, PAMAM and PPI dendrimers, linear polymers, silica, gold and NLC nanoparticles) were screened for inhalation delivery. Preferential accumulation and retention of a carrier, siRNA and drug in the lungs versus other organs after the inhalation delivery were used as criteria for the selection 20, 21, 24, 25, 36, 42. [10-13]As a result of the analysis of our data, we selected a lipid-based as the best carriers for inhalation co-delivery of nucleic acids and drugs. In the present study, we prepared drug-contained positively charged NLC in order to make complexes with negatively charged siRNAs.

Paclitaxel (TAX) was selected as a model lipophilic anticancer drug that can be easily encapsulated in the lipids of NLC. In addition, this drug has been used in the clinic alone or in combination with platinum-based chemotherapeutic agents for treatment of advanced NSCLC 51-55.

A Pool of siRNAs targeted to different TKs was selected to inhibit all four types of EGFRs in NSCLC cells. We found that the high specificity of RNA interference mechanisms and suppression of all four types of EGFRs improved anticancer efficiency of the proposed delivery system and provided a synergetic effect with the anticancer drug. In addition, the pool of siRNAs potentially can expand the range of therapy-responsive NSCLC patients when compared with currently used EGFR-targeted treatment options.

In order to increase the efficiency of treatment of NSCLC and limit adverse side effects on healthy tissues, we are proposing the use of a dual targeting approach. First, the therapeutic exposure is limited mainly to the lungs by the local delivery of nanoparticles by inhalation (passive targeting). Moreover, local inhalation delivery minimizes the destruction of the delivery system during its journey into the circulation and gastrointestinal tract when compared with parenteral and oral routes of administration. Secondly, the distribution of the proposed system in the lungs is shifted toward the preferential accumulation in cancer cells, limiting the toxic exposure of normal non-cancerous lung cells by incorporating of LHRH peptide into the system (active targeting) 20, 37. Such active targeting in turn should enhance the cellular uptake of siRNA and anticancer drug by switching from “simple” endocytosis to receptor mediated endocytosis. Previously we found that receptors to LHRH peptide are overexpressed in many types of cancer cells including human lung cancer cells; in contrast, we did not find a detectable level of the expression of these receptors in normal visceral organs: liver, kidney, spleen, heart, muscle and lung. 9, 10, 13, 20, 22, 56, 57.

Luteinizing Hormone-Releasing Hormone (a previously developed synthetic analog of natural LHRH) was first proposed in our laboratory as a cancer targeting moiety (mostly for targeting of ovarian cancer) and has been studied extensively 9, 10, 12-15, 22, 56. High cancer-targeting ability of LHRH peptide was experimentally confirmed and synthetic procedures for attaching this targeting moiety to different nanocarriers were developed and tested 15-17, 22, 38, 42, 58. The present study showed that targeting of NLC-siRNA-TAX system to lung cancer cells by the LHRH peptide substantially increased the anticancer efficacy of the drug. Data obtained in the present competitive inhibition of cytotoxicity by the LHRH receptor ligand confirm the involvement of LHRH receptors in the internalization of LHRH-targeted nanoparticles and the importance of receptor-mediated endocytosis for cellular internalization of such nanoparticles.

As hypothesized, a novel tumor targeted LHRH-NLC-siRNAs-drug delivery system demonstrated efficient delivery its active payloads (siRNA and TAX) into cancer cells. Proposed dual passive and active targeting approaches (local delivery by inhalation and targeting specifically to lung cancer cells) allowed for a delivery of the highly toxic active components specifically to the lungs with tumor and limiting their accumulation (and possible adverse side effects) in non-tumorous lung tissues and other healthy organs. The efficacy of LHRH-targeted delivery of nanoparticles specifically to the lung cancer cells with limited accumulation in healthy lung tissues was already confirmed in our previous separate study 20. Our data demonstrate that all components of the proposed treatment system (TAX, NLC-siRNAs and non-targeted NLC-siRNAs-TAX) were substantially less effective when compared with complex tumor targeted LHRH-NLC-siRNAs-TAX system. Although such a system is rather complex and its translation to clinical applications may represent some difficulties, the simplification of the system certainly will lead to the decrease in the efficiency of therapy.

## Conclusions

Current investigations demonstrated that the proposed complex delivery system containing five different components led to the suppression of an entire pool of major EGF receptors and substantially enhanced the anticancer activity of paclitaxel to the level that cannot be achieved either by chemotherapy or EGFR-targeted genotherapy alone or by the conventional EGFR-TK inhibitors. Moreover, the proposed approach is almost equally effective in cells with or without mutation of EGFR-TKs. Such effect is normally cannot be achieved by the conventional (antibody or small-molecule TK inhibitors) EGFR-targeted treatment. One also can advocate that the suggested treatment should induce less adverse side effects on healthy organs and normal lung tissues.

## Abbreviations

AALAC: American Association for Laboratory Animal Science
ANOVA: single-factor analysis of variance
DMSO: dimethyl sulfoxide
DOTAP: 1,2-dioleoyl-3-trimethylammonium propane
DSPE: 1,2-distearoyl-sn-glycero-3-phosphoethanolamine
DSPE-PEG: 1,2-distearoyl-sn-glycero-3-phosphoethanolamine-N-[amino(polyethylene glycol)-2000]
EGFRs: epidermal growth factor receptors
IACUC: Institutional Animal Care and Use Committee
IgG: immunoglobulin G
LHRH: luteinizing hormone-releasing hormone
LUAD: lung adenocarcinoma
MRI: magnetic resonance imaging
MTD: maximum tolerated dose
MTT: 3-(4,5-dimethylthiazol-2-yl)-2,5-diphenyltetrazolium bromide
NLCs: nanostructured lipid carriers
NSCLC: non-small cell lung carcinoma
PAMAM: poly(amidoamine)
PBL: peripheral blood lymphocytes
PEG: poly(ethylene-glycol)
PI3K: phosphatidylinositol 3′-kinase
PPI: polypropylene polybenzyl isocyanate
QRT-PCR: real time quantitative reverse transcription polymerase chain reaction
RAS: rat sarcoma
siRNAs: small interfering RNAs
SPC: soybean phosphatidylcholine
STAT: signal transducers and activators of transcription
TAX: paclitaxel
TK: tyrosine kinase
